# Global Predictors of Appointment Non-Adherence in Primary Care Settings: A Systematic Review

**DOI:** 10.3390/healthcare14050623

**Published:** 2026-03-01

**Authors:** Azzam Zohair Jad, Sitah Mohammed Alotaibi, Amjad Mohammed Asiri, Shouq Khalid Alobaid, Rana Ali Algahamdi, Latifa Abdullah Alaqeel, Khamael Fawaz Aljabri, Aisha Khaled Alamer, Jumanah Ibrahim Alsultan, Abdullah Almaqhawi

**Affiliations:** 1 College of Medicine, Umm Al-Qura University, Makkah 24268, Saudi Arabia; s443003076@uqu.edu.sa; 2 College of Nursing, Princess Nourah bint Abdulrahman University, Riyadh 11671, Saudi Arabia; 445010027@pnu.edu.sa; 3 Ministry of Health, Primary Health Care Center, Abha 62523, Saudi Arabia; aasiri448@moh.gov.sa; 4 College of Medicine, Princess Nourah bint Abdulrahman University, Riyadh 11564, Saudi Arabia; 442003468@pnu.edu.sa; 5 College of Medicine, Al-Baha University, Al-Baha 65779, Saudi Arabia; 439003700@stu.bu.edu.sa; 6 College of Medicine, Imam Mohammad Ibn Saud Islamic University, Riyadh 13317, Saudi Arabia; 442022274@sm.imamu.edu.sa; 7 Ministry of National Guard Health Affairs, Al-Ahsa 11426, Saudi Arabia; alamerai1@mngha.med.sa (A.K.A.); alsultanju1@ngha.med.sa (J.I.A.); 8 Department of Family Medicine and Community, College of Medicine, King Faisal University, AlAhsa 31982, Saudi Arabia; aalmuqahwi@kfu.edu.sa

**Keywords:** primary care, appointment adherence, missed appointments, predictors, socioeconomic factors, telemedicine

## Abstract

**Background**: Missed primary care appointments disrupt continuity, reduce care quality, and increase healthcare costs. Despite numerous studies, global patterns and predictors of appointment non-adherence remain inconsistently reported. **Objective**: To identify, categorize, and evaluate the consistency of predictors of appointment non-adherence in primary care across diverse populations and healthcare systems. **Methods**: A systematic review was conducted following PRISMA guidelines (PROSPERO ID: CRD420251121963). PubMed/MEDLINE, Scopus, Web of Science, and Cochrane were searched in August 2025 for observational studies examining predictors of missed, canceled, or rescheduled primary care appointments. Study quality was appraised using the MINORS tool. **Results**: Twenty-seven observational studies (1982–2025) across eight countries were included, representing a total of more than 13 million appointments analyzed. Reported non-attendance varied widely (~5–31%). Predictors clustered into: patient-level (younger age, socioeconomic disadvantage, minority status in North American studies, mental health burden, and lower literacy/greater social needs), appointment-level (prior non-attendance and longer time between booking and visit), and clinic/system-level (access barriers such as transportation and scheduling friction). Telemedicine and continuity with the same clinician were associated with lower non-attendance in more recent studies. Due to heterogeneity in definitions and analyses, the results were synthesized narratively; overall study quality was modest–moderate by MINORS. **Conclusions**: Missed appointments reflect interacting patient- and system-level determinants, with the highest risk among younger and socioeconomically disadvantaged patients and those with mental health conditions. Interventions that reduce access friction (e.g., reminders, flexible scheduling/shorter lead times, transportation support) and equity-focused hybrid telemedicine may reduce non-adherence.

## 1. Introduction

Continuity of care is a cornerstone of primary healthcare, ensuring timely diagnosis, effective management of chronic diseases, and improved long-term outcomes. However, missed appointments, or appointment non-adherence, remain a persistent global issue that disrupts care continuity and contributes to poor health outcomes and inefficient healthcare delivery [1,2,3,4,5]. Non-attendance resulted in delayed treatment, unmet health needs, and significant economic burden on healthcare systems through wasted resources and reduced clinic productivity [3,6,7]. Studies from various regions consistently demonstrate that missed appointments are associated with poorer disease control, increased emergency visits, and higher hospitalization rates, underscoring the clinical and economic importance of addressing this challenge [4,5,6].

The determinants of appointment non-adherence are multifactorial, encompassing patient-level, provider-level, and health system-level factors. Patient-level predictors frequently include sociodemographic characteristics such as age, gender, marital status, employment, income, and education [3,8,9,10,11]. Individuals from socioeconomically disadvantaged backgrounds often experience more barriers to attendance due to financial hardship, transportation limitations, or competing life demands [2,10,12,13]. Psychological factors such as depression, anxiety, and reduced self-efficacy have also been linked to missed visits, with evidence suggesting that mental health burden significantly reduces patients’ engagement in care [14,15,16]. Furthermore, low health literacy and reduced awareness of preventive care have been associated with a lack of motivation to attend scheduled visits [14,15,17]. These findings highlight the need for holistic patient-centered approaches that address both clinical and social determinants of health.

Health system and provider-related factors play an equally critical role in shaping attendance behavior. Inefficient scheduling processes, long waiting times, and limited appointment availability are known to increase the likelihood of missed visits [3,18,19]. Evidence from safety net and family medicine clinics suggests that longer consultation times and more flexible scheduling can reduce future no-shows by improving patient satisfaction and trust [4,18]. Geographic accessibility, including distance to clinics and availability of public transportation, also significantly influences adherence, particularly in rural or underserved regions [2,3,20]. Organizational culture, patient–provider communication, and the use of appointment reminders further affect attendance rates [9,19,21]. A realist review highlighted that effective reminder systems, patient engagement strategies, and supportive communication can mitigate missed appointments when adapted to local contexts [17].

Recent developments in digital health and data analytics have introduced novel strategies for predicting and preventing missed appointments. Machine learning and artificial intelligence models have been increasingly employed to identify patients at high risk for non-adherence by analyzing historical data and behavioral patterns [1,21]. These personalized approaches allow healthcare providers to target interventions such as tailored reminders or outreach calls. By identifying consistent global predictors, such as previous non-attendance and socioeconomic status, the results of this review can be used to directly inform feature selection in predictive models, thus closing the gap between traditional epidemiological research and the application of artificial intelligence. However, the generalizability of such models across countries remains uncertain due to variations in healthcare systems, digital infrastructure, and patient populations [1,2,22]. Similarly, the rise of telemedicine during and after the COVID-19 pandemic has had mixed effects on appointment adherence. While virtual care improved accessibility for some populations, it simultaneously created digital barriers for older adults and low-income groups, emphasizing persistent inequities in healthcare access [23,24,25].

Cultural and contextual differences across regions also shape non-adherence behaviors. Studies from Saudi Arabia, Japan, and Australia have identified unique predictors such as employment obligations, cultural attitudes toward preventive medicine, and regional service delivery structures [3,8,11]. In the United Kingdom and the United States, social deprivation, comorbidity burden, and minority status have been consistently associated with higher rates of missed appointments [4,13,20]. These cross-national variations illustrate that predictors of non-adherence are influenced by broader social and cultural determinants, reinforcing the need for context-specific interventions that align with local health system structures and population needs [3,4,8,11,13,20].

Although the literature on missed appointments has expanded significantly over the past four decades, most studies remain localized, focusing on single populations or healthcare systems. Few have synthesized findings across regions to identify universal and context-specific predictors of appointment non-adherence in primary care [17,21]. The recent increase in studies using advanced analytics and international datasets presents an opportunity to reassess these predictors on a global scale.

In order to address the existing gaps, this systematic review was conducted with a specific research question formulated with a PICO approach as follows: “In patients scheduled for primary care visits globally (P), which patient-, appointment-, and system-related factors (E) are consistently associated with appointment non-adherence when compared with factors associated with attendance (C), and how do these factors relate to appointment non-adherence (O).” Prior evidence suggests that both patient-level characteristics, such as demographics, comorbidities, and psychosocial factors, and system-level characteristics, including scheduling processes and clinic organization, play a role in influencing adherence. Through a systematic review, this study aims to synthesize the global evidence on predictors of appointment non-adherence in primary care.

The objectives of this review were fourfold. First, to identify and categorize the predictors of appointment non-adherence (missed, canceled, or rescheduled appointments) among patients in primary care settings worldwide. Second, to assess the strength and consistency of associations between these predictors and appointment non-adherence across studies and healthcare systems. Third, to evaluate whether predictors differed according to population demographics, healthcare system characteristics, and study regions, such as high-income versus low- and middle-income countries. Finally, to provide a comprehensive evidence base to support the development of targeted interventions and healthcare policies aimed at reducing appointment non-adherence in primary care.

## 2. Methods

In our review, we followed the PRISMA (Preferred Reporting Items of Systematic Reviews and Meta-Analyses) model to minimize selection bias [26]. This study protocol was registered with PROSPERO a priori with the following ID: CRD420251121963. Since the study relied exclusively on previously published data, ethical approval was not required. We conducted a comprehensive search in August 2025 in PubMed/MEDLINE, Scopus, Web of Science, and Cochrane CENTRAL, supplemented by targeted searches and citation chasing. Google Scholar was used as a supplementary source to identify additional records not captured by indexed databases and to support backward/forward citation tracking. Additional studies were identified through manual screening of reference lists of included articles and relevant reviews. The complete database-specific search strategies (all terms/fields and any limits) are provided in Supplementary Materials.

### 2.1. Methodology for Selecting Studies

We included observational studies (cohort, cross-sectional, or case–control), with either retrospective or prospective data, published in English. The population comprised patients attending or scheduled to attend primary care appointments in any healthcare setting worldwide. Eligible studies examined factors associated with appointment non-adherence, rather than those evaluating specific interventions. The comparison of interest was between patients who attended scheduled appointments and those who missed, canceled, or rescheduled them. Studies were required to report statistically analyzed predictors or associated factors of appointment non-adherence.

We excluded studies conducted in secondary, tertiary, specialty, or inpatient care settings and those focusing solely on interventions to improve adherence without analysis of predictive factors. Studies lacking adherence-based stratification or statistical analysis of predictors were also excluded. In addition, editorials, commentaries, reviews, protocols, qualitative-only studies, and case reports were not considered for inclusion. Details of the predefined inclusion and exclusion criteria are summarized in Table 1.

### 2.2. Process of Screening and Data Extraction

One independent reviewer (S.K.) screened papers simultaneously and independently reviewed their title and abstract using the Rayyan search web (https://www.rayyan.ai/, access date: 21 September 2025) and mobile app for systematic reviews [27]. For transparency, title/abstract screening by a single reviewer is a deviation from ideal duplicate screening. However, to ensure the rigor of the selection process and minimize potential bias, full-text eligibility assessment and data extraction were performed in strict duplicate by independent reviewers, representing the more critical stages for ensuring data integrity and study inclusion.

To mitigate selection error, full-text eligibility was assessed by two independent reviewers (A.Z., L.A.), and data extraction was performed in duplicate (J.A., A.A.) with cross-checking to avoid duplication and omissions. General information was collected from the articles, including: author, country, design, sample size, patient age, and year of publication. The target outcomes of missed appointments, canceled appointments, and rescheduled appointments were extracted. In addition, associated risk factors were also extracted. To avoid duplication, the retrieved data were double-checked.

### 2.3. Assessment of Quality and Bias Risk

The included studies were evaluated for methodological quality and risk of bias using the Methodological Index for Non-Randomized Studies (MINORS) [28]. The MINORS tool is a validated 8-item instrument for non-comparative studies and a 12-item instrument for comparative studies, with each item scored 0–2 (maximum scores of 16 and 24, respectively). Two independent reviewers (K.F., R.A.) conducted the assessment; one reviewer performed the initial scoring, and the other verified the results. Discrepancies were resolved through discussion until consensus was reached.

### 2.4. Analytic Plan

A quantitative meta-analysis was not performed due to substantial clinical and methodological heterogeneity, including variability in (i) definitions of non-adherence (no-show vs cancellation vs rescheduling; patient-level vs appointment-level outcomes), (ii) populations and settings (general primary care vs disease-specific cohorts; safety net vs teaching vs national datasets), and (iii) analytic approaches and covariate adjustment across studies. To address this heterogeneity, we conducted a structured narrative synthesis by grouping predictors into patient-level, appointment-level, and health system/provider-level domains, and we summarized whether associations were consistent across regions and health system contexts where data permitted. Predictor consistency was defined as the presence of statistically significant associations in at least three independent studies from two or more distinct geographic regions or healthcare systems.

Given the wide study timeframe (1982–2025), we additionally examined temporal heterogeneity by summarizing predictors across three pragmatic eras reflecting major shifts in care delivery and technology: 1982–2004 (pre–widespread EHR/SMS), 2005–2019 (EHR expansion and routine SMS/automated reminders), and 2020–2025 (telemedicine/COVID-19 era). The specific cut points chosen were intended to be indicative of key inflection points in healthcare technologies and service delivery. The cut point of 2004/2005 marks the period in which electronic health records (EHRs) and SMS reminders were implemented, resulting in a significant impact on appointment management. The cut point of 2020 marks the period in which the COVID-19 pandemic resulted in a rapid and widespread shift in the use of telemedicine and virtual care modalities, thereby fundamentally changing patient access and appointment adherence.

Predictors were interpreted within their historical context, and we prioritized consistency of associations across eras when drawing conclusions.

## 3. Results

### 3.1. Search Results and Study Selection

**Figure 1:**
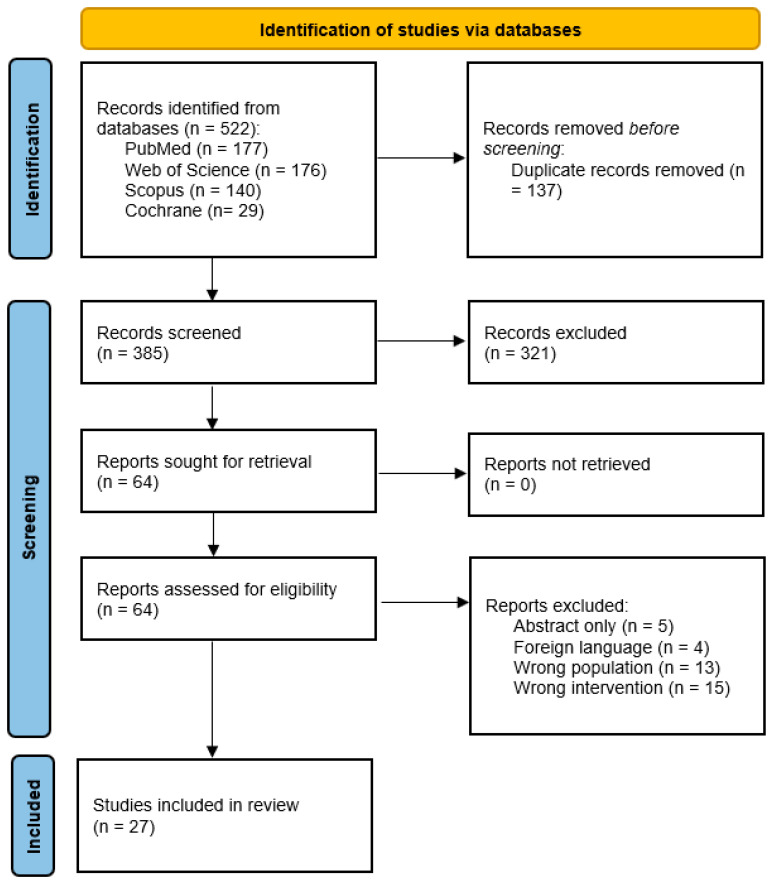
PRISMA flowchart of the screening process.

The PRISMA flow diagram shows that 522 records were identified across four databases, with 137 duplicates removed, leaving 385 records screened. After title/abstract screening, 321 records were excluded, and 64 full texts were assessed for eligibility. Of these, 37 were excluded (mainly due to wrong intervention, population, or abstract-only reports), resulting in 27 studies included in the final review (Figure 1).

### 3.2. Characteristics of Included Studies

Twenty-six studies (1982–2025) from the United States, United Kingdom, Canada, Australia, Argentina, Japan, Thailand, and Saudi Arabia assessed missed primary care appointments across multiple designs (retrospective cohorts, prospective cohorts, cross-sectional analyses, quasi-experiments). Sample sizes ranged from 106 to 824,374 patients, with appointment counts ranging from hundreds to 13.6 million. Populations included general primary care users, diabetes cohorts, HIV clinic patients, and health system-wide samples. Across studies, non-attendance was reported in relation to patient demographics, socioeconomic measures (e.g., Medicaid/uninsured), race/ethnicity and language, mental health measures, health literacy, social needs (including transportation), waiting time, continuity measures, provider seniority, and visit modality (including telemedicine). Summary characteristics of included studies are presented in Table 2 [2,3,4,5,6,7,8,9,10,11,12,14,15,16,18,19,20,23,24,28,29,30,31,32,33,34].

### 3.3. Risk of Bias and Certainty of Evidence

Using the MINORS tool, overall methodological quality was modest to moderate. For non-comparative studies, scores ranged from 6 to 9 (median: 8.5) out of a maximum of 16, while for comparative studies, scores ranged from 12 to 19 (median: 16) out of 24, indicating a moderate level of quality across the included evidence. Common limitations included a lack of prospective sample size calculation, a lack of unbiased endpoint assessment, and incomplete follow-up reporting. Strengths frequently included clearly stated aims, well-defined endpoints, and reporting of patient characteristics (Supplementary Tables S2 and S3).

### 3.4. Prevalence of Missed Appointments

Reported missed appointment rates varied by setting and population. Across studies reporting appointment-level non-attendance percentages, rates ranged from 5.2% to 38.0%, with a median of 14.2%. In large primary care datasets from Western settings, approximately 7–15% of scheduled visits were not attended. For example, an Australian regional clinic reported a 7.6% missed appointment rate over 2 years [3], and a Maine family practice residency reported a 6.7% no-show rate in 1995 [35]. Higher no-show frequencies were reported in some safety net and urban U.S. settings; a Texas Federally Qualified Health Center network reported high volumes of missed visits linked to specific predictors [18], and a New York urban health system reported 26.6% no-shows for primary care appointments [12]. In other settings, a Saudi Arabian primary care audit reported a 29.5% default rate [8], and an Argentinian clinic reported approximately 23% non-attendance [9].

### 3.5. Patient Demographic Factors

Age: Across studies, younger adults more frequently had higher non-attendance than older adults. Weingarten et al. reported the highest no-shows among 17–30-year-olds in a family practice clinic [32], and Neal et al. reported decreasing odds of missing with increasing age group in UK general practice [20]. Some studies also reported higher non-attendance among very elderly patients (e.g., >80–90 years) [4]. A Thai diabetes study reported patterns consistent with work and responsibility-related constraints in middle-aged adults [10]. One study reported high kept appointment rates for infants and higher missed rates among adolescents [29].

Gender: Findings were mixed across studies. Some studies reported slightly higher non-attendance among women, including a modestly higher odds estimate in an Australian clinic, with subgroup variation among Aboriginal women [3,36]. Other studies reported limited or no independent association after adjustment; for example, in an HIV clinic analysis, gender was not a key predictor after accounting for poverty and age [13]. In some settings, subgroup patterns were reported (e.g., self-employed men) [3,11].

Race/ethnicity and language: Several North American studies reported higher no-show rates among racial/ethnic minority groups. Goldman (1982) reported higher non-attendance among non-White patients [28]. Adepoju et al. reported higher odds of missed visits among Black patients compared with White patients in Texas safety net clinics [18], and other studies reported differences across racial/ethnic groups, including Black and Hispanic patients. Smith et al. reported differences in appointment keeping by race/ethnicity in a Midwestern clinic [31]. Telemedicine studies reported changes in disparities by race/ethnicity [23]. In Saudi Arabian settings, ethnicity was not a reported variable, while education level was reported as a predictor in that context [8]. Language-related differences were reported in a U.S. study of missed appointments by preferred language, including Spanish, Portuguese, and Haitian-Creole speakers [24].

Socioeconomic status: Low socioeconomic status was frequently associated with higher non-attendance across multiple measures (income/poverty, insurance type, education, area deprivation indices). A Scottish analysis reported deprivation as a strong correlate of repeated missed appointments [4]. In U.S. studies, Medicaid and uninsured status were commonly associated with higher no-show rates [18,32]. Medicaid managed care enrollment was also associated with higher missed appointments in one study [31], and uninsured status was reported as a predictor of poorer retention in HIV care [34]. Educational attainment was reported as a predictor in a U.S. analysis linking EHR to neighborhood measures [2], and limited reading ability was associated with non-attendance in one study [14]. Some studies reported differing patterns by payer categories, including self-pay versus Medicaid, depending on the setting [29]. Area-level deprivation measures were also reported as predictors in U.S. clinic data [2].

### 3.6. Psychosocial and Health-Related Factors

Mental health: Multiple studies reported associations between mental health measures and missed appointments. Depression screening positivity was associated with missed visits in one study [14]. In a psychiatry referral context, lower distress was associated with non-attendance [16]. Ciechanowski et al. reported complex patterns of healthcare utilization and missed visits among patients with major depression [15]. McQueenie et al. reported higher mortality risk among patients with frequent missed appointments, with stronger associations among those with mental health conditions [6].

Interpersonal factors and beliefs: Ciechanowski et al. reported associations between attachment style and missed primary care visits [15]. Non-completion of depression screening was associated with attendance outcomes in one study [16]. Studies also reported patient-reported reasons for missing appointments, such as issue resolution or perceived lack of necessity [20]. Patient satisfaction was not predictive in Goldman’s study [28].

Comorbidity and chronic disease: McQueenie et al. reported associations between the number of long-term conditions and missed appointments, including interactions with mental health conditions [6]. Diabetes cohorts showed variable non-attendance rates and reported associations with outcomes such as glycemic control and hospitalization [7]. Prior engagement measures (e.g., number of prior visits) were reported as predictors in some studies [18].

Personality and social support: One study reported exploratory findings relating personality traits (e.g., agreeableness) to missed appointments [10]. Neal reported patterns of reattendance after missed appointments in UK general practice [20].

### 3.7. Healthcare System and Appointment-Related Factors

Lead time: Longer time between scheduling and the appointment date was associated with higher non-attendance in multiple studies [9,32]. In psychiatry referrals, longer wait time was associated with lower attendance [30]. Same-day appointments were reported to have higher keep rates in one study [29]. McQueenie et al. discussed appointment timing in relation to missed visits [6].

Timing: Several studies reported associations between no-shows and appointment time slots or days of the week, including afternoon clinics [8] and patterns by weekday [3,9].

Reminders: Patient-reported reasons for missing appointments included forgetting to cancel or attend [19]. Reminder interventions were reported in a Saudi pain clinic context, with a change in no-show rates after phone reminders [37]. Studies varied in how reminder systems were described.

Transportation and distance: Transportation was reported as a barrier in studies linking social needs and missed appointments and in survey-based reporting [8,12]. Distance-to-clinic measures were included as predictors in multiple settings, with varying reported associations [18,29]. Geographic and rurality-related measures were included as predictors in a U.S. analysis [2]. Telehealth was evaluated as a visit modality in a post-2020 study [23].

Visit type, continuity, and provider type: Studies reported associations between visit purpose (preventive/routine versus other visits), continuity with clinicians, and provider type/seniority and attendance [18,20,29,32]. Teaching settings reported variation by provider training level [32].

Cancellation and rescheduling processes: Studies reported that patients sometimes intended to cancel but were unable to do so [20], and non-attenders frequently did not cancel in advance [19]. Some studies reported higher cancellation volumes relative to no-shows and associations between rescheduling patterns and subsequent care utilization [31].

Telemedicine and post-2020 studies: Telehealth was evaluated as a visit modality in post-2020 primary care studies, including subgroup analyses by race/ethnicity and payer categories [23]. Additional interventions reported included targeted reminder programs [37] and an attendance promotion program in a diabetes cohort [11]. Overbooking was noted as an administrative approach in clinical practice but was not a primary outcome in the included studies.

### 3.8. Outcomes and Consequences of Missed Appointments

Several studies reported associations between missed appointments and clinical outcomes or healthcare utilization. Hwang et al. reported that higher no-show propensity was associated with incomplete screening and poorer disease control, as well as higher emergency department and hospitalization use [5]. In diabetes populations, missed visits were reported in relation to HbA1c and hospitalization outcomes [37], with post-hospitalization missed visits linked to rehospitalization risk in one study [7]. McQueenie et al. reported associations between frequent missed appointments and higher mortality, with stronger associations among patients with mental health conditions [6]. Neal reported that many patients who missed appointments later reattended within a short period [20]. Economic outcomes were reported, including NHS cost estimates related to missed GP appointments [38,39] and clinic revenue impacts in a U.S. safety net context [24]. Training and workflow consequences were also described in residency settings [32].

### 3.9. Temporal Patterns in Predictors (1982–2025)

Studies were additionally summarized by study period (pre-2000, 2000–2019, post-2020) and health system context (e.g., tax-funded vs insurance-based systems) in Table 3. Across periods, studies reported consistent inclusion of demographic, socioeconomic, and prior attendance measures, while later-period studies more frequently evaluated EHR-derived predictors, automated reminders, and telemedicine-related factors.

## 4. Discussion

This review synthesized evidence from 27 studies (1982–2025) examining predictors of missed primary care appointments across multiple countries and care contexts. To support interpretation across diverse settings, we apply a multi-level framework in which non-adherence reflects the joint influence of patient-level factors, provider relationships, clinic processes, and wider system conditions.

Across studies, younger age, socioeconomic disadvantage, and prior non-attendance were repeatedly reported correlates of missed appointments [4]. Several studies also reported associations with race/ethnicity and language in North American settings [18,24,29] and with mental health measures in multiple contexts [6,14,15,30]. For instance, the absence of ethnicity as a reported variable in Saudi Arabian contexts, where the level of education was found as a predictor [8], further emphasizes that constructs of disparity are highly contextual and show considerable regional variation.

Collectively, these findings indicate that non-attendance commonly clusters with markers of social and clinical vulnerability.

Appointment adherence should be interpreted within the context of access and service design rather than as an isolated patient attribute. Repeated non-adherence can be framed as a marker of system–patient misalignment and structural constraint, rather than patient failure [39]. Studies addressing attachment style and patient attitudes also reported associations consistent with the relevance of trust, continuity, and perceived value of care [15]. Follow-up approaches described in included studies highlight the role of proactive outreach for patients who miss visits, particularly in groups with higher clinical risk [6]. Mental health factors may contribute to missed visits through amotivation, disorganization, avoidance, and symptom burden, which may interact with logistical barriers.

Structural barriers were frequently reported, including transportation needs [8,12], work constraints, long lead times [9,32], and difficulty canceling or rescheduling [19,20]. Clinic-level process variables (lead time, reminder systems, cancellation ease, and scheduling flexibility) were repeatedly included as predictors and may differ across health systems and care models.

Telemedicine was evaluated in post-2020 studies and was associated with lower no-show rates in primary care, including subgroup patterns by race/ethnicity and payer category [23].

At the same time, telemedicine has limitations and ethical considerations, including digital exclusion related to device access, connectivity, digital literacy, sensory/cognitive limitations, privacy constraints, and appropriateness for conditions requiring physical examination. Apart from these challenges, there are some ethical issues specific to algorithmic bias in access platforms, where there might be an unintentional bias against vulnerable populations if such systems were developed using non-representative data [39,40]. There are also some new privacy issues, such as data breaches during virtual consultations and ensuring patient privacy in shared living spaces [41,42]. In order to address these challenges, new “equity-aware hybrid” models should be implemented, which include integrating proactive digital literacy screening, providing “digital navigators” for vulnerable populations, and auditing these new technologies for algorithmic bias. They should also ensure an uninterrupted experience between virtual and face-to-face care, so that there is no compromise in care quality for vulnerable populations who might be excluded from these new technologies [43,44].

### 4.1. Limitations

Most included studies were observational, limiting causal inference. Study settings, outcome definitions, and analytic adjustments varied substantially, contributing to heterogeneity. The long inclusion window introduces temporal heterogeneity; therefore, some predictors (e.g., transportation barriers) may not be directly comparable across decades. Publication bias is possible. Finally, heterogeneity limited quantitative pooling; therefore, the results were synthesized narratively with emphasis on predictors repeatedly reported across contexts.

### 4.2. Future Directions

The synthesized predictors support hypothesis-driven intervention testing. Examples include: (1) a high-risk bundle for patients with prior missed appointments and socioeconomic disadvantage (rapid rebooking + navigator/peer outreach + transport support) compared with standard reminders; (2) friction reduction scheduling for younger adults (short lead times, simplified cancellation/rescheduling, and waitlist backfill) compared with usual scheduling; and (3) hybrid telehealth with digital support for older or low-digital-literacy patients compared with telehealth alone.

Predictive analytics using EHR data has also been evaluated in included studies and may enable targeted outreach based on prior attendance and risk profiles [2,4]. Additional work should assess longer-term outcomes associated with reducing missed appointments, including utilization and mortality endpoints [6]. (4) Policy-level interventions for addressing systemic barriers include the following. In addition to clinic-level and patient-level interventions, there is a need for policy-level interventions for mitigating systemic determinants of non-adherence. It is important for policymakers and payers to explore different payment mechanisms for increasing the length of clinic appointments, especially for patients who are socioeconomically disadvantaged, as studies indicate that longer appointment times can prevent future missed appointments [18]. Furthermore, insurance coverage can also be designed to provide for non-medical determinants of health, such as transportation services, which can help address issues of access as identified in studies [8,12]. Such policy interventions acknowledge non-adherence as a symptom of systemic misalignment with patients and work towards a more equitable healthcare system.

## 5. Conclusions

Missed primary care appointments were associated with multiple patient-level and system-level factors across included studies. Predictors frequently reported included younger age, socioeconomic disadvantage, prior non-attendance, and mental health measures, alongside clinic process factors such as lead time, reminders, and cancellation/rescheduling pathways. Future research should prioritize targeted, testable interventions aligned with identified risk profiles and local health system context, with attention to equity and access in both in-person and telehealth care.

## Figures and Tables

**Table 1:** PICOS criteria for inclusion and exclusion of the study.

PICO Element	Inclusion Criteria	Exclusion Criteria
Population	Patients attending or scheduled to attend primary care appointments across any global healthcare setting.	Patients seen in secondary, tertiary, specialty care, or inpatient settings.
Exposure/predictors	Factors associated with appointment non-adherence.	Studies focused on interventions to reduce non-adherence without analyzing predictors.
Comparison	Patients who attended scheduled appointments versus those who missed them.	No comparison group or studies that did not stratify results by adherence status.
Outcomes	Predictors or associated risk factors of missed, canceled, or rescheduled appointments.	Studies that did not report statistically analyzed predictors or associated factors.
Study designs	Observational studies (cohort, cross-sectional, or case–control), with either retrospective or prospective data.	Editorials, commentaries, reviews, protocols, case reports, or qualitative-only studies.

**Table 2:** Characteristics of the included studies.

Study (First Author, Year)	Country	Design	Number of Participants and Appointments	Males (Number, Percent)	Age (Summary)	Non-Attendance Prevalence (Definition)	Key Positive/Negative Predictors
Goldman, 1982 [28]	United States (Boston, Massachusetts)	Prospective patient interview with six-month chart follow-up in a hospital primary care clinic	376 patients; 1181 non-canceled appointments	122 (32%)	Median 56 years	18% (appointment level)	Higher risk with younger age, non-White race, clinician-identified psychosocial problems, and prior missed visits
Al-Shammari, 1991 [8]	Saudi Arabia (Riyearsadh)	Audit of primary care clinics in two university hospitals	3292 patients	Not reported	Patients older than 12 years; defaults are highest at 65 years or older	29.5% (appointment level)	Older age, afternoon, and late-week booking were associated with higher default rates
Smith, 1994 [29]	United States (Minnesota)	Family practice residency clinic records with targeted chart audit and logistic regression	4669 patients; 7283 physician appointments	Not reported	Attendance increased with age; best in patients 60 years or older	26.1% (appointment level)	Higher risk with Hispanic or African American race, traditional Medicaid insurance, Friday or Saturday scheduling; same-day visits were better kept
Grunebaum, 1996 [30]	United States (New York, New York)	Retrospective chart review of referrals for psychiatric consultation in a primary care clinic	180 referred patients (90 attended; 90 missed in analytic sample)	49 (27.2%)	Mean about 47 years	38% (appointment level)	Higher risk with mild distress, resistance to referral, and longer wait from referral to appointment
Majeroni, 1996 [31]	United States (Buffalo, New York)	Retrospective cohort in an urban family care center with multivariable analysis	477 patients; 2772 scheduled appointments	Not reported	Highest miss rates in 19–25 years; lowest in children and adults older than 55 years	48% (patient level)	Medicaid managed care coverage had roughly double the risk compared with other insurance types after adjustment
Weingarten, 1997 [32]	United States (Maine)	Family practice residency clinic over 36 sampled days; chi-square and two-way analysis of variance	3962 appointments	Not reported	Attendance increased with age; lowest in 17–30 years; highest older than 64 years	6.7% (appointment level)	Higher risk with Medicaid and self-pay compared with private or Medicare; postgraduate year 1 residents had higher non-attendance than faculty; no difference by sex
Neal, 2005 [20]	United Kingdom (West Yorkshire)	Postal questionnaire of adults who missed a general practice appointment with three-month record review	386 who missed (122 respondents) and 386 matched attenders	Not reported	Adults; odds of missing decreased with age	NR	Common reasons: forgot, could not cancel or inconvenient time, family demands or illness; prior misses predicted future missing
Ciechanowski, 2006 [15]	United States (Washington State)	Cross-sectional survey linked to automated electronic health record data in nine health maintenance organization clinics with one-year follow-up	3923 patients	2020 (51.5%)	Mean about 63 years	Patient level: 13.8%; appointment level: 4.1%	Variation by visit type; depression and attachment style explored
Nuti, 2012 [7]	United States (Indiana)	Prospective cohort of adults with diabetes; Andersen–Gill months of six-month emergency department visits and hospitalizations	8787 patients	3519 (40.1%)	Adults older than 18 years; the probability of missing decreased with age	16.2% (appointment level)	Higher subsequent hospitalization only among those with a previous six-month admission; preventable diabetes admissions were months frequent after missed visits
Giunta, 2013 [9]	Argentina (Buenos Aires)	Retrospective cohort of adult general medicine outpatients with predictive modeling from administrative and electronic records	44,402 patients; 170,574 requested appointments	16,809 (37.9%)	Adults older than 18 years	22.7% (appointment level)	Higher risk with prior missed visits, longer wait time, multiple same-day bookings; more likely after 5:00 pm and in June, July, and December
Nancarrow, 2014 [3]	Australia (New South Wales)	Retrospective cohort of scheduled primary care appointments with multivariable logistic regression	8634 patients; 90,785 analyzed appointments	4166 (48.8%)	Median 43 years (appointment level)	7.6% (appointment level)	Higher risk with younger age, fewer prior appointments, and Aboriginal or Torres Strait Islander status; higher on Monday and Friday
Miller-Matero, 2016 [14]	United States (Detroit, Michigan)	Health system chart review of consecutive primary care psychology patients	147 patients	53 (36.1%)	Mean 52.5 years	15.4% (patient level)	Independent predictors: probable depression and limited reading ability
Hwang, 2015 [5]	United States (Boston, Massachusetts)	Large primary care network: developed a no-show propensity factor from five-years history	140,947 patients	About 42–45% across groups	Mean age decreased from low- to high-propensity groups	Group mean non-attendance: 2.4% (low), 17.8% (intermediate), 31.8% (high)	Higher propensity predicted incomplete screening, poorer disease control, and higher emergency department and hospitalization use
Ellis, 2017 [4]	United Kingdom (Scotland)	National retrospective cohort using routine general practice appointment data from 2013 to 2016	550,083 patients; 13,623,316 appointments	260,714 (47.4%)	Mean 45 years (interquartile range 25–61)	12.1% (appointment level); 46% (patient level)	Persistent individual behaviour with repeated missing patterns linked to deprivation and long-term conditions
McComb, 2017 [33]	United States (Indiana)	Prospective cohort of adults with diabetes across 20 outpatient clinics; outcomes after attended, canceled, or missed index appointment	7586 patients; 46,710 appointments	Not reported	Adults older than 18 years	17.7% (patient level) 12.2% (appointment level)	Rescheduling after the index appointment was associated with longer delay and higher emergency department use; missed appointments with after-index rescheduling were worst
Kay, 2019 [34]	United States (Southeast)	Single-site HIV clinic electronic medical record analysis with multinomial logistic regression	1159 patients	835 (72.0%)	Mean 44 years (range 20–83)	Any missed appointment 39.5% (31.2% one to two; 8.3% three or more)	Higher risk with younger age, poverty, lack of insurance, less than high school education, and lack of Ryan White HIV/AIDS Program support; poverty distinguished frequent missers
McQueenie, 2019 [6]	United Kingdom (Scotland)	National data linkage of general practice appointments to death registry; follow-up to 2017	824,374 patients; 11,490,537 appointments	About 383,458 (46.5%)	Population across age groups	NR	All-cause mortality rose stepwise with more missed visits; strongest in people with long-term mental health conditions
Claveau, 2020 [19]	Canada (Quebec)	Observational study in four family medicine teaching units; retrospective records plus patient survey	34,619 scheduled appointments; 1757 survey respondents	Not reported (survey about 28% male)	Younger than 50 years were more likely to miss	7.8% (appointment level)	Lower non-attendance with physicians and nurses than residents; seasonal peaks; common reasons were issues resolved, work, and inconvenient timing
Fiori, 2020 [12]	United States (Bronx, New York)	Health system social needs screener linked to electronic health records in 19 primary care practices	41,637 patients	16,698 (40.1%)	Highest non-attendance among 18–29 and 30–44 years; lowest 65 years or older	26.6% (patient level)	More social needs increased risk in a dose–response manner; transportation need was the strongest
Hayashino, 2010/2011 [16]	Japan	Prospective observational analysis from a cluster trial pilot in primary care for type 2 diabetes; depression screening and later attendance	1444 patients; 1409 person-years of follow-up	Not reported	Mean about 55 years	6.3% (patient level)	Non-completion of depression screening was associated with higher risk; depressive symptoms alone were not significant
Nakayama, 2022 [11]	Japan	Secondary analysis of a cluster trial of adults with type 2 diabetes; sex-specific Cox models	2010 patients; 2068 person-years	1249 (62.1%)	Median 58 years (interquartile range 53–61)	men 70.4 and women 43.1 per 1000 person-years	Among men, self-employment increased risk, and an adherence promotion intervention reduced risk; no employment effect in women
Jirmanus, 2022 [24]	United States (Massachusetts)	Difference-in-differences analysis of electronic health records in two safety net systems before and after immigration policy changes	159,054 patients; 806,411 primary care visits	71,613 (45.0%)	All ages	Missed appointments increased from 20.1% to 21.0% overall	Increase was larger among Spanish, Portuguese, and Haitian-Creole speakers than among English speakers
Shah, 2023 [23]	United States (Arizona)	Retrospective cohort across two primary care networks; comparison of telemedicine with office visits	164,647 patients; 311,517 visits	Not reported	Adults 18 years and older	Telemedicine had 5.2% no-show compared with 7.3% for office visits (appointment level)	Telemedicine reduced disparities most for Black and Hispanic or Latino patients and for Medicaid and self-payer insurance
Sae-Ueng, 2024 [10]	Thailand	Cross-sectional descriptive study with questionnaire and medical records in a primary care unit	106 patients	37 (34.9%)	Median 65 years (interquartile range 58–75)	Self-reported: 32.1% never missed, 28.3% occasionally missed, 39.6% regularly missed (patient level)	Descriptive only; no multivariable model reported
Tuan, 2024 [2]	United States (Pennsylvania)	Retrospective cross-sectional analysis linking electronic health records with socioeconomic and geographic data across 14 clinics	75,186 patients; 258,590 appointments	32,615 (43.4%)	Highest risk in 18–39 years; lowest 80 years or older	7.8% (appointment level)	Higher risk with minority race or ethnicity, Medicaid insurance, lack of insurance, non-English language, greater distance, and neighborhood deprivation; better continuity and longer physician tenure reduced risk
Adepoju, 2025 [18]	United States (Texas)	Retrospective cohort in a federally qualified health center network using electronic health records	28,090 patients; 56,180 appointments	About 10,112 (36%)	Adults mainly 18–64 years (87%); 6% 65 years or older	NR	Higher risk among uninsured patients and some minority groups; longer prior appointment duration was linked to fewer future missed visits

NR: not reported.

**Table 3:** Categorization of included studies by study period and health system type, with corresponding dominant predictors.

Study Period	Health System Context	Predictors Most Consistently Associated with Non-Attendance
Pre-2000	Mixed	Younger age, long waits, limited cancellation options
2000–2019	Insurance-based/tax-funded	SES disadvantage, prior misses, mental health
Post-2020	Mixed	Telemedicine (protective), digital access, SES gradients

## Data Availability

No new data were created or analyzed in this study.

## Supplementary Materials

The following supporting information can be downloaded at: https://www.mdpi.com/article/10.3390/healthcare14050623/s1, Table S1. Search strategy. Table S2. MINORS assessment tool for non-randomized non-comparative studies. Table S3. MINORS assessment tool for non-randomized comparative studies.
