# Supplementary material for: Global Predictors of Appointment Non-Adherence in Primary Care Settings: A Systematic Review
Source: Healthcare (Basel). 2026 Mar 1;14(5):623. doi: 10.3390/healthcare14050623 (PMC12984880; doi:10.3390/healthcare14050623)
Supplement: Supplementary file 1 [file healthcare-14-00623-s001.zip › healthcare-4129578-supplementary.pdf]

healthcare MDPI

Supplementary tables

Database	Search Query	Re-sults
PubMed	(“missed appointment*” OR “no-show*” OR “failed attendance” OR “appointment nonattendance”) AND (“primary care” OR “family medicine” OR “general practice”) AND (predictor* OR “risk factor*” OR determinant* OR adherence OR compliance)	177
Scopus	TITLE-ABS-KEY (“missed appointment*” OR “no-show*” OR “failed attendance” OR “appointment nonattendance”) AND (“primary care” OR “family medicine” OR “general practice”) AND (predictor* OR “risk factor*” OR determinant* OR adherence OR compliance)	140
Web of science	(“missed appointment*” OR “no-show*” OR “failed attendance” OR “appointment nonattendance”) AND (“primary care” OR “family medicine” OR “general practice”) AND (predictor* OR “risk factor*” OR determinant* OR adherence OR compliance) (Topic)	176
Cochrane	(“missed appointment*” OR “no-show*” OR “failed attendance” OR “appointment nonattendance”) AND (“primary care” OR “family medicine” OR “general practice”) AND (predictor* OR “risk factor*” OR determinant* OR adherence OR compliance)	29

Supplementary Table S1. Search strategy

 https://doi.org/10.3390/healthcare14050623

Item	Tuan WJ, 2025	Tuan WJ, 2024	Natsuda Sae-Ueng, 2024	Fiori KP, 2020
1. A clearly stated aim	2	2	2	2
2. Inclusion of consecutive patients	2	1	1	1
3. Prospective collection of data	0	0	0	0
4. Endpoints appropriate to the aim of the study	2	2	2	2
5. Unbiased assessment of the study endpoint	2	2	0	2
6. Follow-up period appropriate to the aim of the study	1	1	1	2
7. Loss to follow-up less than 5%	0	0	0	0
8. Prospective calculation of the study size	0	0	0	0
Study total	9	8	6	9

Supplementary Table S2. MINORS assessment tool for non-randomized non-comparative studies

Item	Goldman., 1982	McComb., 2017	Ellis., 2017	McQueenie .,2019	Claveau., 2020	Kay., 2019	Adepoju OE., 2025	Shah DA., 2023	Jirmanus LZ., 2022	Nakayama.,2022	McComb et al., 2017	Ellis et al., 2017
1. A clearly stated aim	2	2	2	2	2	2	2	2	2	2	2	2
2. Inclusion of consecutive patients	1	2	2	2	1	1	1	1	1	1	1	1
3. Prospective collection of data	0	2	0	0	0	0	0	0	0	0	1	0
4. Endpoints appropriate to the aim of the study	2	2	2	2	2	2	2	2	2	2	2	2
5. Unbiased assessment of the study endpoint	1	1	1	1	1	1	2	2	2	2	0	0
6. Follow-up period appropriate to the aim of the study	1	2	2	2	1	2	2	1	2	2	1	1

Item	Kay et al., 2019	McQueenie et al., 2019	Fiori et al., 2020	Claveau et al., 2020	Hayashino et al., 2011 (J-DOIT2)	Jirmanus et al., 2022	Nakayama et al., 2022	Shah et al., 2023	Sae-Ueng & Luvira, 2024	Tuan et al., 2024	Adepoju et al., 2025	Tuan et al., 2025
1. A clearly stated aim	2	2	2	2	2	2	2	2	2	2	2	2
2. Inclusion of consecutive patients	2	2	2	2	2	1	2	1	2	2	2	2
3. Prospective collection of data	1	2	2	2	1	1	1	1	2	2	2	2
4. Endpoints appropriate to the aim of the study	2	2	2	2	2	2	2	2	2	2	2	2
5. Unbiased assessment of the study endpoint	0	0	0	0	0	0	0	0	0	0	0	0
6. Follow-up period appropriate to the aim of the study	2	2	2	2	2	1	1	2	2	2	2	2

**7. Loss to follow-up less than 5%**	0	1	0	0	0	0	0	0	0	0	0	0	1	1	1	1	0	0	0	1	1	1	1	1
**8. Prospective calculation of the study size**	0	0	0	0	0	0	0	0	0	0	0	0	0	0	0	0	0	0	0	0	0	0	0	0
**9. An adequate control group**	1	1	1	1	1	1	1	2	2	1	2	2	2	2	2	2	2	2	2	2	2	2	2	2
**10. Contemporary groups**	2	2	2	2	2	2	2	2	2	2	1	1	2	2	2	2	1	1	2	2	2	2	2	2
**11. Baseline equivalence of groups**	1	1	1	1	1	1	1	1	1	1	1	1	2	2	2	2	2	1	2	2	2	2	2	2
**12. Adequate statistical analyses**	1	2	2	2	2	2	2	2	2	2	2	2	2	2	2	2	2	2	2	2	2	2	2	2
**Total Score**	12	18	15	15	13	14	15	15	16	15	13	12	18	19	19	19	16	13	16	17	19	19	19	19

Supplementary Table S3. MINORS assessment tool for non-randomized comparative studies

https://doi.org/10.3390/healthcare14050623
